# Src activity is modulated by oxaliplatin and correlates with outcomes after hepatectomy for metastatic colorectal cancer

**DOI:** 10.1186/1471-2407-14-660

**Published:** 2014-09-10

**Authors:** Scott Kopetz, Van K Morris, Nila Parikh, Michael J Overman, Zhi-Qin Jiang, Dipen Maru, Paul Elvin, Gary Gallick

**Affiliations:** Department of Gastrointestinal Medical Oncology, The University of Texas MD Anderson Cancer Center, Houston, TX USA; Department of Cancer Medicine, The University of Texas MD Anderson Cancer Center, Houston, TX USA; Department of Genitourinary Medical Oncology, The University of Texas MD Anderson Cancer Center, 1515 Holcombe Boulevard, Unit 0018-4, Houston, TX 77030 USA; Department of Pathology, The University of Texas MD Anderson Cancer Center, Houston, TX USA; AstraZeneca, Wilmington, Delaware USA

**Keywords:** Colorectal cancer, Metastasis, Hepatectomy, Src oncogene, Focal adhesion kinase

## Abstract

**Background:**

The nonreceptor tyrosine kinase Src regulates multiple pathways critical to tumor proliferation, chemoresistance, and epithelial-to-mesenchymal transition. It is robustly activated after acute oxaliplatin exposure and in acquired oxaliplatin resistance *in vitro* and *in vivo*, but not after 5-fluorouracil (5-FU) alone. However, activation of Src and its substrate focal adhesion kinase (FAK) in metastatic colorectal cancer treated with oxaliplatin has not been investigated. We retrospectively evaluated the activation of Src and FAK in hepatic metastases of colorectal cancer and correlated these findings with the clinical outcomes of patients treated with oxaliplatin.

**Methods:**

Samples from 170 hepatic resections from patients with metastatic colorectal cancer from two cohorts were examined by IHC for expression of Src, activated Src (pSrc), FAK, and activated FAK (pFAK). Patients in the first cohort (120 patients) were analyzed for immunohistochemical protein expression and for survival outcomes. In the second cohort, tissue was collected from 25 patients undergoing sequential hepatic metastasectomies (n = 50).

**Results:**

In the first cohort, Src activation was positively correlated with pFAK expression (*P* = 0.44, *P* < 0.001). Patients pretreated with oxaliplatin and 5-FU demonstrated increased expression of pFAK (*P* = 0.017) compared with patients treated with 5-FU alone or irinotecan/5-FU. Total Src expression was associated with the number of neoadjuvant cycles of oxaliplatin (*P* = 0.047). In the second cohort, pFAK expression was higher following exposure to oxaliplatin. When patients were stratified by expression of pFAK and pSrc, an inverse relationship was observed between relapse-free survival rates and levels of both pFAK (21.1 months, 16.5 months, and 7.4 months for low, medium, and high levels of pFAK, respectively; *P* = 0.026) and pSrc (19.6 months, 13.6 months, and 8.2 months, respectively; *P* = 0.013). No differences in overall survival were detected.

**Conclusions:**

Patients administered neoadjuvant oxaliplatin demonstrated higher levels of Src pathway signaling in hepatic metastases, a finding associated with poorer relapse-free survival. These results are consistent with prior *in vitro* studies and support the idea that combining Src inhibition with platinum chemotherapy warrants further investigation in metastatic colorectal cancer.

**Electronic supplementary material:**

The online version of this article (doi:10.1186/1471-2407-14-660) contains supplementary material, which is available to authorized users.

## Background

The 60-kDa nonreceptor tyrosine kinase Src is among the nine members of the Src family kinases, which regulate cell proliferation, migration, adhesion, invasion, differentiation, and angiogenesis [1–3]. While Src is found throughout all human cells [4], increased activity of this proto-oncogene has been described in multiple solid tumors, including breast, lung, pancreatic, and ovarian cancers [5]. Cellular stimuli induce conformational changes that increase Src kinase activity via dephosphorylation of residue Y530 and autophosphorylation of residue Y416 [6]. Interaction of activated Src with adjacent signaling enzymes and cytoskeletal proteins subsequently triggers multiple downstream pathways such as PI3K/Akt and Ras/Raf/MAPK, which have been implicated in tumor survival and proliferation [7].

In colorectal cancer, overexpression of the Src protein and epigenetic changes in the tumor cell have been correlated with increased activity of Src kinase [3, 8], which clinically has been associated with shorter disease-free survival in patients undergoing curative resection and with shorter overall survival in patients with metastatic disease [9]. Src hyperactivity also has been associated with a more aggressive tumor phenotype through promotion of epithelial-to-mesenchymal transition [10] and through focal adhesion kinase (FAK)-mediated tumor cell motility [11]. In addition, *in vivo* studies have shown that Src activity is higher in colorectal cancer cells relative to adjacent normal colonic epithelium [12], as well as in hepatic metastases relative to the primary colorectal tumor [13].

*In vivo* studies for multiple solid tumors have demonstrated that Src activity is implicated in resistance to chemotherapy and that inhibition of Src may restore sensitivity to chemotherapy [14–16]. Although no convincing benefit for Src inhibitors as single agents in patients with refractory metastatic colorectal cancer has thus far been reported from early-phase trials [17], preclinical studies have shown that the combination of the Src inhibitor dasatinib with oxaliplatin significantly reduced the volume of hepatic metastases in mice relative to treatment with either agent alone [18]. When given in combination with 5-FU, Src was robustly activated after acute oxaliplatin exposure and in acquired oxaliplatin resistance *in vitro* and *in vivo*, but not after 5-FU alone.

Activation of Src (denoted by phosphorylation at Y416) and its substrate FAK (phosphorylated at Y861) in metastatic colorectal cancer treated with oxaliplatin has thus far not been investigated. The purpose of this study was to retrospectively assess 170 samples from hepatic metastases of patients with colorectal cancer to determine the activation of Src and FAK when treated with platinum-based chemotherapy. We assessed the implication of protein/activated protein levels on clinical outcomes. We found that neoadjuvant oxaliplatin was associated with higher levels of FAK activation in hepatic metastases compared with non-oxaliplatin-based regimens, a finding associated with poorer relapse-free survival. These results are consistent with those of prior *in vitro* studies correlating oxaliplatin exposure with activation of the Src pathway and support the idea that combining inhibition of Src with platinum chemotherapy warrants further investigation in patients with metastatic colorectal cancer.

## Methods

### Patient demographics

The MD Anderson institutional computerized database was reviewed in order to collect information regarding the clinical characteristics and pathologic features of tumors from patients studied in this series. Chi-squared analysis was performed to assess for differences in gender distribution for the various cohorts, and Fisher’s exact t-tests were employed to compare distributions of patients according to ethnicity, site of primary tumor, histological subtype, and tumor grade.

### Tissue procurement

Two separate cohorts of patients were used for these retrospective studies. No tissue was collected prospectively. Records at our institution were screened to identify patients with metastatic colorectal cancer treated between 12/1995 and 9/2011 who had undergone resection for hepatic metastases. Patients analyzed either presented at initial diagnosis with metastatic disease or developed metastatic disease following a prior resection of the primary tumor. Patients with no remaining tumor available for immunohistochemical staining were excluded from consideration.

In the first cohort, tissue from 120 patients with metastatic colorectal cancer was collected from hepatic metastectomy. These patients either received no neoadjuvant treatment, or received combination treatment with either 5-fluorouracil and oxaliplatin (FOLFOX) or 5-fluorouracil and irinotecan (FOLFIRI) prior to hepatectomy. From each tissue block, tumor-bearing regions were elected and placed into a tissue microarray, with the coefficient of variation within the same resected sample (% CV_within_) calculated to estimate the intraspecimen variation. If the % CV_within_ (representative of technical variability) was greater than the coefficient of variation between (% CV_between_) individual patients (representative of biologic variability), then the biopsies were flagged for reanalysis; in most cases, one core was a clear outlier and removed from further analysis.

In the second cohort, metastases removed from 25 patients who underwent sequential hepatic resections (constituting 50 cases) were collected in a tissue microarray, with two tumor cores and one normal liver core per case. These patients were free of disease after the first hepatic resection and then had a second resection for recurrent liver-limited disease. They had received no chemotherapy before the first resection; between the two resections, they had either received no chemotherapy, or received treatment with either oxaliplatin (FOLFOX) or irinotecan (FOLFIRI). All tissue was collected, stored, and studied under approval of the Institutional Review Board at MD Anderson.

### Immunohistochemical analysis

**Figure 1:**
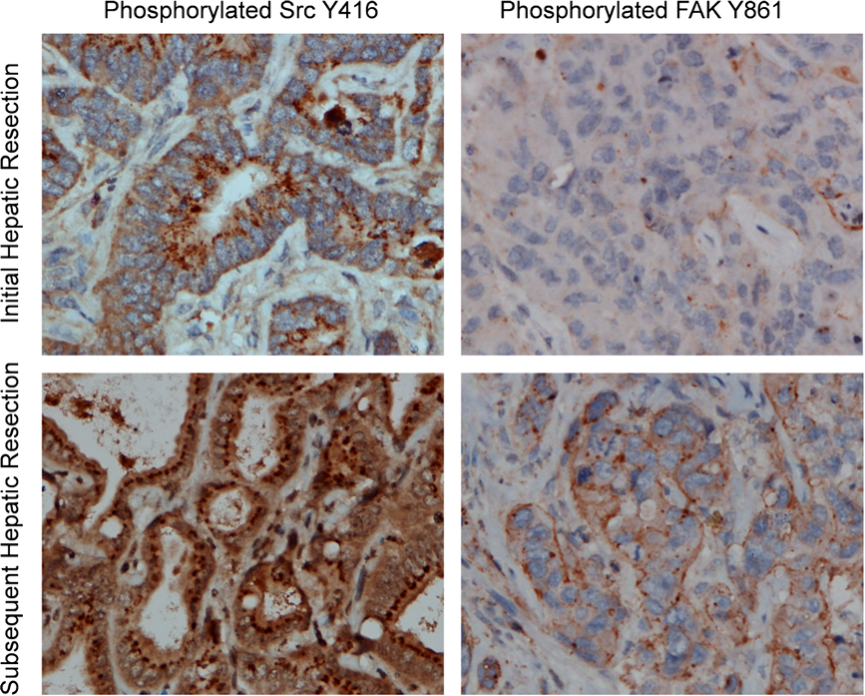
**Immunohistochemistry for pSrc and pFAK in hepatic metastases from colorectal cancer.** The left panels demonstrate pSrc (Y416) staining in one patient before and after treatment with oxaliplatin chemotherapy (top and bottom, respectively). In the right panels, pFAK (Y861) staining demonstrates a similar pattern.

Paraffin-embedded tissue was sliced at 8 μm thickness prior to mounting. Paraffin was removed by heating of the slides to 60°C for 30 minutes followed by placement in a bath of xylene followed by a series of increasingly diluted ethanol bath. For staining of Src and phosphorylated forms of Src, the slides were boiled in a pressure cooker for 5 minutes at 125°C in a bath of Borg decloaker soluation (Biocare Medical Inc.). For staining of FAK and phosphorylated forms of FAK, the slides were placed in a bath of EDTA buffer and boiled in a microwave oven for 5 minutes, followed by treatment with Dako target retrieval solution (Dako North America, Inc.) for one hour. Peroxidase activity was blocked by incubation with 3% hydrogen peroxide for 12 minutes. The slides were rinsed with PBS for 3 minutes each for 3 times, followed by a protein block solution (Cyto Q immune-diluent buffer; Innovex) for 20 minutes at room temperature. Antibodies were diluted in the protein block solution at the specified dilution ratio in a volume of 50 to 100 μL and incubated at 4°C overnight. A negative control was incubated in protein block solution without the primary antibody added. Slides were then washed again in PBS (3 minutes × 3) followed by treatment with the secondary antibody (Mach 4 Universal HRP polymer, Biocare Medical Inc, or 4 + Goat anti-rabbit biotinylated antibody, Biocare Medical Inc.). Seventy microliters of diaminobenzidine (DAB) was applied for 2–10 minutes followed by rinsing after sufficient staining was developed. Counterstaining was done with Gill’s No. 3 hematoxylin (Sigma), followed by drying and mounting with Universal mount (Open Biosystems). Mounted slides were visualized using a bright field microscope.Primary antibodies utilized were anti-Src antibody (1:100, Cell Signaling Technology), anti-phospho-Src family kinase Y416 (1:100 to 1:500, Cell Signaling Technology), anti-FAK antibody (1:100, Cell Signaling Technology), anti-phospho-FAK Y861, antibody (1:100, BioSource/Invitrogen), and anti-PTEN (DAKO). Tissue was stained with antibodies for Src, pSrc, FAK, pFAK, and PTEN. Regions containing tumor were identified using manual masking of tumor-bearing regions by an Ariol automated scanning microscope and image analysis system. Specimens were next measured for quantitative protein expression with a DAB filter. After normalization of total protein levels, immunohistochemical stains were visualized at 20× objective and were graded by automated quantitating image analysis (Aperio Technologies, Vista, CA; U.S.A.) using a score of 0, 1+, 2+, or 3+ according to membrane staining intensity and completeness of the tested biomarker. This same scoring system was used for both sets of tissue microarrays and thereby allows for a concordance in scoring between the two series (Figure 1). The stains were compared by the Mann–Whitney test (first cohort) or paired Wilcoxon signed-rank test (second cohort). Both the investigators performing the tumor masking and the pathologist interpreting the staining were blinded to prior treatments of the patients whose tumors were analyzed. Pearson’s correlation coefficients were calculated to assess the relationship between levels of total Src, pSrc, total FAK, and pFAK.

### Mutational analysis

Samples from the first cohort of patients were analyzed to determine whether levels of Src and FAK expression were associated with particular mutations. DNA was extracted using a QIAamp DNA FFPE tissue kit (Qiagen, Valencia, CA; U.S.A.) from formalin-fixed, paraffin-embedded tissue taken from whole mounts of the same blocks used for tissue microarray analysis. DNA was next sequenced using Sequonom MassArray mass spectrometry technology (Sequonom, Inc., San Diego, CA, U.S.A) to assess for mutations in particular point mutations including but not limited to *KRAS*, *NRAS*, *BRAF*, *PIK3CA*, and *CTNNB1* (see Additional file 1: Table S1 for full listing of genes sequenced). Metastatic samples harboring a particular mutation were compared against colorectal tumors lacking these mutations to assess for differences in levels of protein expression.

### Survival analysis

Patients from the first cohort of 120 patients were grouped into three groups according to levels of activated protein as quantified by immunohistochemical staining. Those falling within one standard deviation of the mean were classified in the “medium” expression group, and those outside this range were deemed to have “high” or “low” expression. Relapse-free survival and overall survival were calculated by group according to Kaplan-Meier methodology.

## Results

### Patient demographics

Among the 120 patients in the first cohort to undergo hepatic metastectomy, 62 had received no chemotherapy prior to resection, 20 had been treated with neoadjuvant FOLFIRI, and 38 with neoadjuvant FOLFOX regimens. Demographic features and clinicopathologic characteristics were similar between the three groups (see Additional file 2: Table S2). Patients undergoing chemotherapy received a mean 7.4 cycles and waited an average of 10.6 weeks after their last dose of chemotherapy before proceeding to surgery. On average, 2.2 liver metastases (range 1–13) were resected, with the mean diameter of the largest metastasis measuring 2.7 cm (range 0.8-10.5 cm). No statistically significant differences for any of the above characteristics were detected between recipients of FOLFOX and FOLFIRI.

Among the 25 patients in the second cohort, 8 received 5-FU chemotherapy between sequential hepatic resections, while 9 patients and 8 patients were treated with irinotecan-based and oxaliplatin-based chemotherapy regimens, respectively. Patients in this cohort received a mean 7.9 cycles (range 1–12) between surgeries. On average, there were 1.48 (range 1–9) hepatic implants removed at the second surgery, with a mean size in maximum diameter of 2.0 cm (range 0.3-5.0 cm). Of note, no differences in patient characteristics were seen between the two cohorts of 120 and 25 patients studied (see Additional file 2: Table S2).

### Tissue microarray quality

Table 1 lists the distribution of inevaluable cases, single core biopsies analyzed, and double core biopsies analyzed for each of the four proteins among the 120 patients in the first cohort. Some cases were inevaluable because tissue sample was lost during preparation, and other biopsies could not be used because insufficient tumor was detected in the sample. For all four antibodies used (Src, pSrc, FAK, and pFAK), the coefficient of variation between samples was greater than the coefficient of variation of the two core biopsies within an individual tumor (Table 1).

**Table 1:** **Measures of tissue microarray quality (cohort 1, N = 120)**

	Inevaluable core biopsies	Single core biopsies	Double core biopsies	CV _between_	CV _within_
FAK	4	18	98	12.8%	6.4%
pFAK	12	29	79	17.4%	9.8%
Src	4	16	100	16.9%	7.4%
pSrc	5	18	97	14.1%	6.3%

Key: CV_between_: coefficient of variation between mean values of the 120 individual patients; CV_within_: coefficient of variation between the two core biopsies within a single patient’s tumor.

### Immunohistochemical staining for Src, FAK, and activated products

Correlations between Src, FAK, pSrc, and pFAK in the first cohort of patients are listed in Table 2. A strong correlation was seen between pFAK and total Src (R = 0.520, *P* < 0.001), as well as between pFAK and pSrc (R = 0.438, *P* < 0.001). Likewise, a strong correlation was measured between total Src and pSrc (R = 0.692, *P* < 0.001), although no correlation was noted for total FAK with either Src or pSrc. No correlations were observed between expression of FAK, Src, and their activated products and the number of hepatic metastases, size of metastases, sex of the patient, or age of the patient.

**Table 2:** **Pearson’s correlation coefficients between mean values of antibody staining for FAK, pFAK, Src, and pSrc (cohort 1, N = 120)**

	FAK	pFAK	Src	pSrc
FAK	—	0.084	0.178	0.171
pFAK	0.084	—	0.520	0.438
Src	0.178	0.520	—	0.692
pSrc	0.171	0.438	0.692	—

**Figure 2:**
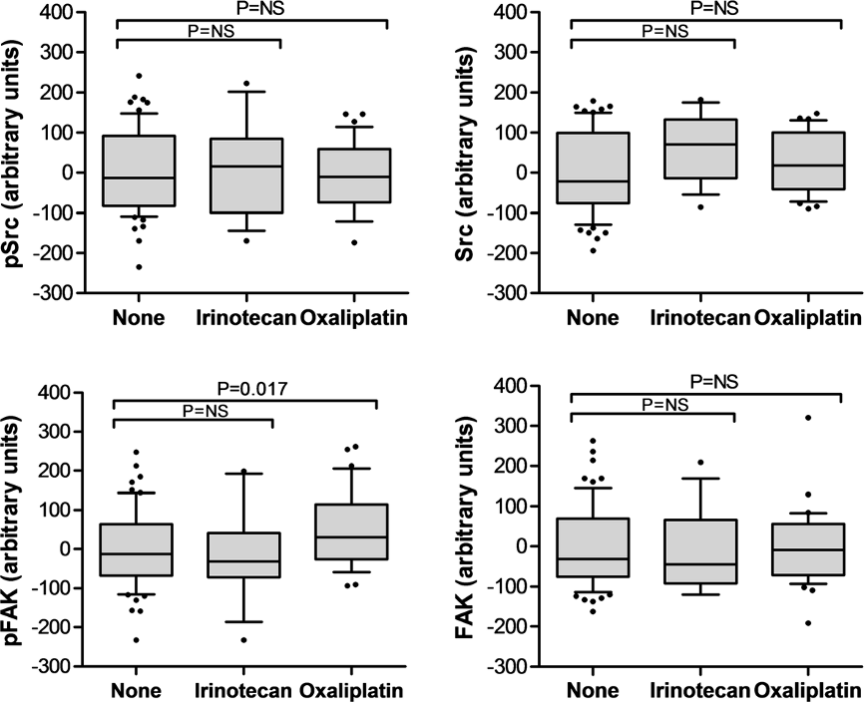
**Levels of pSrc, Src, pFAK, and FAK in liver metastases treated with various chemotherapeutic regimens.** Patients exposed to oxaliplatin demonstrated higher levels of pFAK expression when compared to patients exposed to a regimen containing irinotecan or 5-FU alone (cohort 1, N = 120).

In the first cohort, in patients treated with oxaliplatin, expression of pFAK in the metastases was elevated compared to expression in untreated patients (*P* = 0.017, Figure 2). Total FAK was unchanged. There was no increase in pSrc after oxaliplatin, although there was a nonsignificant trend toward increased total Src after oxaliplatin chemotherapy. Total Src expression was correlated with the number of cycles of chemotherapy administered (*P* = 0.047).

**Figure 3:**
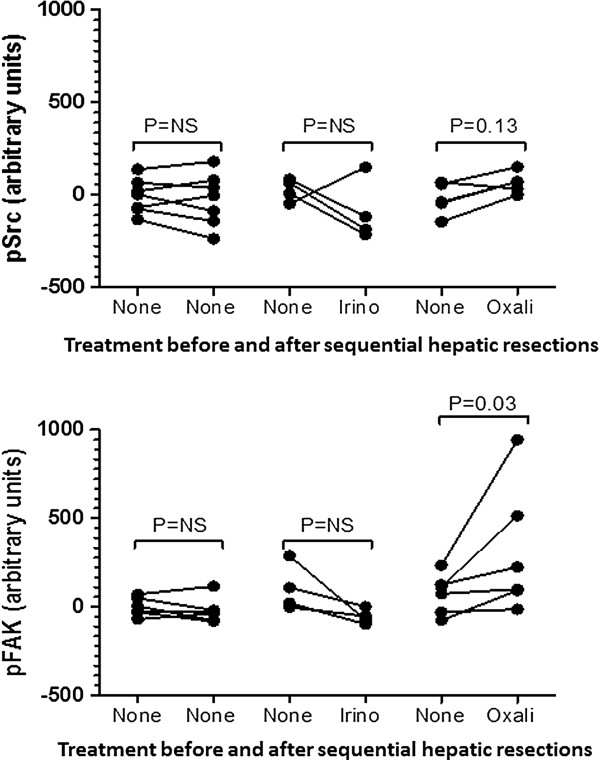
**Analysis of pSrc and pFAK expression in sequential liver metastases resected from the same patients.** Patients were treated with no chemotherapy before the first resection and either with an oxaliplatin-based regimen, an irinotecan-based regimen, or no chemotherapy between resections (cohort 2, N = 15 for pSrc and N = 16 for pFAK). Each pair of points represents a single patient. Patients exposed to oxaliplatin demonstrated an increased in activated pFAK and a trend towards increased pSrc that was not observed in the patients who received no chemotherapy or an irinotecan-based regimen.

A second cohort of 25 patients who underwent two sequential hepatic metastectomies was analyzed for differences in activated Src and FAK expression in the separate tumor specimens. Fifteen cases had sufficient tissue available to compare pSrc expression, and sixteen patient samples were evaluated to compare pFAK expression. While Src phosphorylation was quantitatively increased after oxaliplatin, the increase did not reach statistical significance (*P* = 0.13, Figure 3). However, the levels of pFAK were significantly higher following exposure to platinum chemotherapy (*P* = 0.03), but unchanged after irinotecan chemotherapy or between surgical samples in which patients were not exposed to cytotoxic chemotherapy.

### Correlation of Src and FAK with gene mutations and PTEN expression

Sequenom analysis was used to detect mutations for KRAS, NRAS, beta-catenin, BRAF, and PIK3CA in the first cohort. No relationship between the presence of *KRAS*, or *BRAF* mutations and activated Src or FAK was appreciated. Ten samples were found to have NRAS mutations, and nine samples contained beta-catenin mutations; six samples carried both. For patients with NRAS-mutant tumors, decreased proportions of pSrc/Src (0.94 vs. 0.74, *P* = 0.006) and pFAK/FAK (1.01 vs. 0.55, *P* < 0.001) were detected relative to their wild-type tumors (see Additional file 3: Table S3); likewise, significantly lower ratios of pSrc/Src (0.94 vs 0.69) and pFAK/FAK (1.01 vs. 0.56) were associated with beta-catenin mutations (*P* < 0.001 for both). Levels of PTEN protein expression were not significantly correlated with FAK, Src, pFAK, and pSrc when quantified using immunohistochemical analysis, although a trend between PTEN expression and total FAK expression was observed (P = 0.054).

### Survival outcomes according to pSrc and pFAK expression

**Figure 4:**
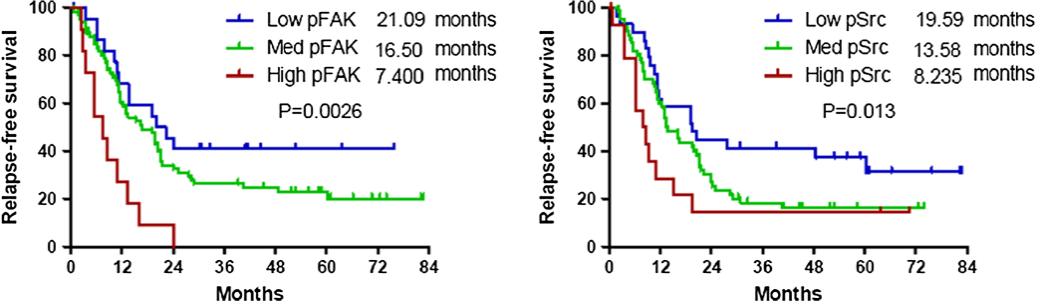
**Relapse-free survival according to relative expression of pFAK and pSrc (cohort 1, N = 120).** Patients with higher levels of pFAK and pSrc, respectively, demonstrated shorter periods of relapse-free survival.

**Figure 5:**
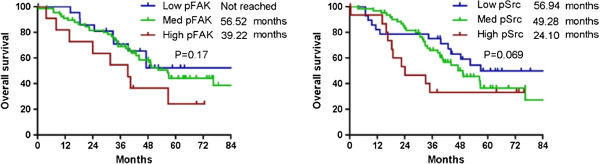
**Overall survival according to relative expression of pFAK and pSrc (cohort 1, N = 120).** A non-significant trend towards worse overall survival was noted in those patients with higher levels of pFAK and pSrc.

In the first cohort, median relapse-free survival were 21.1 months, 16.5 months, and 7.4 months, for the low-, medium-, and high-pFAK-expression groups, respectively (*P* = 0.003, Figure 4); similarly, median relapse-free survival durations were 19.6 months, 13.6 months, and 8.2 months for low, medium, and high expression levels of pSrc, respectively (*P* = 0.013). No significant differences in overall survival were noted among the three expression-level groups for either pFAK or pSrc (Figure 5).

## Discussion

In this study, evidence of upregulated signaling of the Src pathway was observed in hepatic metastases after oxaliplatin-based chemotherapy among patients with colorectal cancer. Not only did we detect this correlation for oxaliplatin (and not for irinotecan or for 5-FU only based regimens) within a cohort of 120 patients treated with different neoadjuvant therapies prior to resection of liver metastases, but we also confirmed these results in an additional cohort of 25 patients who underwent sequential hepatic metastasectomies. In both groups, higher levels of pFAK, the activated target of Src, were seen after exposure to oxaliplatin. Prior *in vivo* work has demonstrated increased Src phosphorylation in mice after treatment with oxaliplatin. Our findings are the first to confirm this effect in human specimens, here among resected liver metastases. In addition, increased Src signaling, with pFAK expression levels as a surrogate, are associated with worse relapse-free survival. The results generated here raise the hypothesis that pFAK may serve as a prognostic biomarker in the future for patients being treated for metastatic colorectal cancer.

Here, we show a strong correlation in tissue samples between activated FAK and expression of both total Src and activated Src. The only recognized mediator of FAK phosphorylation at Y861 is Src, so this epitope is an excellent, specific indicator of Src kinase activity. The demonstration in our study of a tight correlation of Src activity and pFAK (P < 0.00001) supports the clinical relevance of this relationship. The performance of the pFAK antibody provided greater dynamic range and staining properties compared with the other three antibodies tested, suggesting that it would be an optimal biomarker for future studies of Src activity. In contrast, the antibody recognizing Y416 on Src also recognizes the corresponding epitope on other members of the Src family -- such as Fyn, Lyn, and Yes – which can be overexpressed in colon cancer [19–21]. Activated Src may serve as an inferior, less-sensitive biomarker for Src signaling in colorectal cancer specimens.

Prior *in vitro* data have shown that oxaliplatin activates Src via reactive oxygen species intermediates and that Src activation is a mechanism of oxaliplatin resistance [18]. These experiments prompted the exploration of the relationship between oxaliplatin administration and activation of signal transduction pathways in human specimens of metastatic colorectal cancer. We show in two cohorts that patients treated with oxaliplatin have tumors with higher levels of activated pFAK relative to untreated patients or patients administered other neoadjuvant regimens. There was a trend toward increased total Src expression with extended duration of oxaliplatin treatment in the larger cohort, but changes in pSrc did not reach statistical significance. While this discordance is likely due to antibody specificity, there may also be differences in the sensitivity of the two different phosphorylated residues to phosphatases present during the fixation process or differences in the temporal dynamics of chronic Src and FAK activation after the several-week chemotherapy washout prior to surgery [22].

Because the use of freshly fixed paraffin tumor has technical limitations, we took steps to minimize the impact of these concerns, including optimization of reagents and staining conditions, and use of freshly cut sections for the respective tissue microarrays. In similar experiments, phosphoepitopes have been shown to be conserved in formaldehyde-fixed, paraffin-embedded tissue, with phosphorylated Akt levels using similar methodology studied as markers for PI3K/Act activation in metastatic colorectal tissues [23]. In addition, we recognize that these results should not be yet generalized to all metastatic colorectal carcinomas, as our studies were performed only in liver metastases.

Specimens were also assessed for any association between protein/activated protein levels and various mutations. No association was found for either *KRAS* or *BRAF* – two genes that, when mutated, have important therapeutic and/or prognostic implications in metastatic colorectal cancer; however, statistically significant lower Src and FAK activity was detected in the presence of *NRAS* and *CTTNB1* mutations (*P* < 0.01 for all). The clinical implication of this association is unclear, as *NRAS* mutations in metastatic colorectal cancer have not been associated with relevant clinical or pathologic features in prior population-based studies among patients treated with oxaliplatin [24]. In our cohort, both *NRAS* (N = 10) and *CTTNB1* (N = 9) mutations were rare, and six patients harbored both mutations. However, in a larger set at our institution of 246 patients with metastatic colorectal cancer with only mutation data available, a pattern of dual *NRAS*/*CTTNB1* mutations could not be confirmed.

Activation of Src has been implicated as a mechanism of cytotoxic chemotherapy resistance in human pancreatic cell lines [15]. When grouped according to relative levels of pFAK expression, patients with higher levels of pSrc and pFAK were noted to have a shorter recurrence-free survival (*P* = 0.013 and *P* = 0.0026, respectively). Similarly, a trend toward a shorter overall survival was noted in the patients with greater expression of the activated kinases. Although statistical significance was not reached, neoadjuvant oxaliplatin has been shown to upregulate Src pathway activation, and any detriment in overall survival caused by activation of these signal transduction pathways in generating chemoresistance may be confounded by the survival benefit of neoadjuvant oxaliplatin in the treatment of metastatic colorectal cancer [25]. Therefore, relapse-free survival may serve as a more informative endpoint when using pSrc and pFAK as biomarkers in the future. Nonetheless, while the immediate clinical applicability of these markers is limited, these results provide a useful insight into the biology of liver metastases and should be considered in future attempts to develop a patient selection marker for this population.

## Conclusions

In summary, prior *in vitro* studies have shown that cellular stress arising from reactive oxygen species following oxaliplatin exposure upregulate Src activation and that chronic Src activation may be one mechanism of resistance to oxaliplatin in metastatic colorectal cancer. Here we report that Src signaling is elevated in patients administered oxaliplatin for neoadjuvant treatment of metastatic colorectal cancer metastases. These findings affirm the importance of Src-mediated signal transduction in the biology of chemoresistant colorectal cancer and suggest that blocking this pathway with Src inhibitors warrants continued consideration in clinical trials.

## Electronic supplementary material

Additional file 1: Table S1: List of specific point mutations assessed by Sequonom MassArray mass spectrometry. (DOCX 17 KB)

Additional file 2: Table S2: Associations (A) between the cohorts of patients studied in the two microarrays and (B) according to the preoperative chemotherapy administered for patients in the first cohort reveal no differences in patient characteristics. (DOCX 16 KB)

Additional file 3: Table S3: Associations between mutation status and relative levels of activated Src and FAK. (DOCX 14 KB)

## Abbreviations

5-FU: 5-fluorouracil
FAK: Focal adhesion kinase
pSrc: phosphorylated Src
pFAK: phosphorylated focal adhesion kinase
FOLFOX: 5-fluorouracil and oxaliplatin
FOLFIRI: 5-fluorouracil and irinotecan
CV: Coefficient of variation
PBS: Phospho-buffered saline.
