# Supplementary material for: Src activity is modulated by oxaliplatin and correlates with outcomes after hepatectomy for metastatic colorectal cancer
Source: BMC Cancer. 2014 Sep 10;14:660. doi: 10.1186/1471-2407-14-660 (PMC4167273; doi:10.1186/1471-2407-14-660)
Supplement: Supplementary file 1 — Additional file 1: Table S1: List of specific point mutations assessed by Sequonom MassArray mass spectrometry. (DOCX 17 KB) [file 12885_2014_4839_MOESM1_ESM.docx]

**Additional file 1: Table S1: List of specific point mutations assessed by Sequonom MassArray mass spectrometry**

| \| AKT1_E17K_G49A \| \| --- \| \| AKT1_G173R_G517C \| \| AKT1_K179M_A536T \| \| AKT2_E17K_G49A \| \| AKT2_G175R_G523C \| \| AKT3_E17K_G49K \| \| AKT3_G171R_G511A \| \| ALK_1174I_T3520A \| \| ALK_A877S_G2G29T \| \| ALK_D1091N_G3271A \| \| ALK_F1245C_T3734G \| \| ALK_F1245V_T3734G \| \| ALK_I1171N_T3512A \| \| ALK_I150T_T3749C \| \| ALK_L560F_G1680C \| \| ALK_M1166R_T3497G \| \| ALK_R1275Q \| \| BRAF_D594_1781A \| \| BRAF_E586K_1756GA_SPLICE \| \| BRAF_G466_1397G \| \| BRAF_G466R_1396_GC \| \| BRAF_K601E_AG \| \| BRAF_K601N_A \| \| BRAF_L597R_1790TG \| \| BRAF_V600_1798G_1 \| \| BRAF_V600_1799T_1 \| \| BRAF_V600_1799T_2 \| \| BRAF_V600_1800G \| \| CDK4_R24C_C70T_2 \| \| CDK4_R24H_G71A \| \| CTNNB1_D32_94G \| \| CTNNB1_D32_95A \| \| CTNNB1_G34_101G \| \| CTNNB1_S33_97T \| \| CTNNB1_S37_109T \| \| CTNNB1_S37_110C \| \| CTNNB1_S45_133T \| \| CTNNB1_S45_134C \| \| CTNNB1_T41_121A \| \| EGFR_G719_G2155 \| \| EGFR_K860I_A2579T \| \| EGFR_L858R_TG \| \| EGFR_S720P_T2158C \| \| EGFR_T790M_C2369T_Splice \| \| EGFR_T854I_C2561T \| \| EGFR_Y813C_A2438G \| \| FRAP_M135T_T404C \| \| GNAS_Q227_C651 \| \| GNAS_R201_C601 \| \| IDH1_R132_C394T \| | \| IDH1_R132_G295T \| \| --- \| \| IDH2_R172_A514T \| \| IDH2_R172_G515 \| \| IGF1R_A1347V_C40404T \| \| JAK2_V617F_G1849T \| \| KIT_D816H_GC \| \| KIT_D816V_AT \| \| KIT_K642E_AG \| \| KIT_L576P_TC \| \| KIT_N556D_AG \| \| KIT_R634W_CT \| \| KIT_V559_T \| \| KIT_V560D_TA_SPLICE \| \| KIT_V825A_TC \| \| KIT_Y553N_TA \| \| KRAS_A146_436 \| \| KRAS_G10R \| \| KRAS_G12_34G \| \| KRAS_G12_35G \| \| KRAS_G13_37G \| \| KRAS_G13_38G \| \| KRAS_Q61_181C \| \| KRAS_Q61_182A \| \| KRAS_Q61_183A \| \| MEK1_D67N_G119A \| \| MET_H1112Y_C3334T \| \| MET_H1124D_C3370G \| \| MET_M1268T_T3803C \| \| MET_N375S \| \| MET_N848S \| \| MET_R988C \| \| MET_T1010I_C3029T \| \| MET_Y1248_T3742 \| \| MET_Y1248C_A3743G \| \| MET_Y1253D_T3757G \| \| NRAS_G12_G34 \| \| NRAS_G12_G35 \| \| NRAS_G13_G37 \| \| NRAS_G13_G38 \| \| NRAS_Q61_A182 \| \| NRAS_Q61_A183 \| \| NRAS_Q61_C181 \| \| PDGFRA_D842_A2525T \| \| PDGFRA_D842_G2524 \| \| PDGFRA_N659K_C1977A \| \| PDGFRA_N659Y_A1975T \| \| PDGFRA_V561D_T1682A \| \| PDGFRA_V824L_G2470C \| \| PDPK1_D527E_C1581G \| \| PDPK1_T354M_C1061T \| | \| PHLPP2_L1016S_T3047C \| \| --- \| \| PIK3CA_A1046V \| \| PIK3CA_C420R \| \| PIK3CA_C420R_2 \| \| PIK3CA_E110K \| \| PIK3CA_E418K \| \| PIK3CA_E453K \| \| PIK3CA_E542_1624G \| \| PIK3CA_E542_1625A \| \| PIK3CA_E545_1633G \| \| PIK3CA_E545_1634A \| \| PIK3CA_E545_1635G \| \| PIK3CA_F909L \| \| PIK3CA_H1047 \| \| PIK3CA_H1047_1 \| \| PIK3CA_H1047Y \| \| PIK3CA_H701P \| \| PIK3CA_K111N \| \| PIK3CA_M1043I_G3129 \| \| PIK3CA_M1043V \| \| PIK3CA_N345K \| \| PIK3CA_P539R \| \| PIK3CA_Q060K \| \| PIK3CA_Q546_1636C \| \| PIK3CA_Q546_1637A1 \| \| PIK3CA_R088Q \| \| PIK3CA_S405F \| \| PIK3CA_Y1021_3061T \| \| PIK3CA_Y1021C_3062 \| \| PIK3R1_D560Y \| \| PIK3R1_intron1 \| \| PIK3R1_intron2 \| \| PIK3R1_M326I_G978 \| \| PIK3R1_N564K \| \| PRKAG1_R70Q \| \| PRKAG2_N488I \| \| RET_M918T \| \| Rictor_M675I_G2025A \| \| Rictor_S159F_C476T \| \| STK11_D194_A591T \| \| STK11_D194_G590 \| \| STK11_F354L_C1062G \| \| STK11_P281L_C842T \| \| TNK2_E346K_G1036A \| \| TNK2_R99Q_G296A \| |
| --- | --- | --- | --- | --- | --- | --- | --- | --- | --- | --- | --- | --- | --- | --- | --- | --- | --- | --- | --- | --- | --- | --- | --- | --- | --- | --- | --- | --- | --- | --- | --- | --- | --- | --- | --- | --- | --- | --- | --- | --- | --- | --- | --- | --- | --- | --- | --- | --- | --- | --- | --- | --- | --- | --- | --- | --- | --- | --- | --- | --- | --- | --- | --- | --- | --- | --- | --- | --- | --- | --- | --- | --- | --- | --- | --- | --- | --- | --- | --- | --- | --- | --- | --- | --- | --- | --- | --- | --- | --- | --- | --- | --- | --- | --- | --- | --- | --- | --- | --- | --- | --- | --- | --- | --- | --- | --- | --- | --- | --- | --- | --- | --- | --- | --- | --- | --- | --- | --- | --- | --- | --- | --- | --- | --- | --- | --- | --- | --- | --- | --- | --- | --- | --- | --- | --- | --- | --- | --- | --- | --- | --- | --- | --- | --- | --- | --- | --- |
