# Supplementary material for: Src activity is modulated by oxaliplatin and correlates with outcomes after hepatectomy for metastatic colorectal cancer
Source: BMC Cancer. 2014 Sep 10;14:660. doi: 10.1186/1471-2407-14-660 (PMC4167273; doi:10.1186/1471-2407-14-660)
Supplement: Supplementary file 2 — Additional file 2: Table S2: Associations (A) between the cohorts of patients studied in the two microarrays and (B) according to the preoperative chemotherapy administered for patients in the first cohort reveal no differences in patient characteristics. (DOCX 16 KB) [file 12885_2014_4839_MOESM2_ESM.docx]

**Additional file 2: Table S2:** Associations (A) between the cohorts of patients studied in the two microarrays and (B) according to the preoperative chemotherapy administered for patients in the first cohort reveal no differences in patient characteristics.

(A)

|  | **Cohort 1**  **(N=120)** | **Cohort 2**  **(N=25)** | ***P-value*** |
| --- | --- | --- | --- |
| Gender |  |  | 0.19 |
| Male | 62 | 17 |  |
| Female | 58 | 8 |  |
| Ethnicity |  |  | 0.88 |
| Caucasian | 96 | 19 |  |
| African-American | 17 | 4 |  |
| Hispanic | 5 | 1 |  |
| Asian | 2 | 1 |  |
| Site of Primary Tumor |  |  | 0.36 |
| Right Colon | 24 | 2 |  |
| Left Colon | 62 | 15 |  |
| Rectum | 34 | 8 |  |
| Tumor Histology |  |  | 0.20 |
| Mucinous | 20 | 1 |  |
| Signet Ring | 2 | 0 |  |
| Neither | 98 | 24 |  |
| Grade |  |  | 0.83 |
| Well differentiated | 1 | 0 |  |
| Moderately differentiated | 89 | 20 |  |
| Poorly differentiated | 18 | 5 |  |
| Unknown | 12 | 0 |  |

(B)

|  | **Oxaliplatin**  **(N=38)** | **Irinotecan**  **(N=20)** | **None (N=62)** | ***P-value*** |
| --- | --- | --- | --- | --- |
| Gender |  |  |  | 0.35 |
| Male | 17 | 21 | 36 |  |
| Female | 21 | 11 | 26 |  |
| Ethnicity |  |  |  | 0.47 |
| Caucasian | 29 | 15 | 52 |  |
| African-American | 3 | 0 | 2 |  |
| Hispanic | 5 | 4 | 8 |  |
| Asian | 1 | 1 | 0 |  |
| Site of Primary Tumor |  |  |  | 0.21 |
| Right Colon | 6 | 3 | 15 |  |
| Left Colon | 20 | 8 | 34 |  |
| Rectum | 12 | 9 | 13 |  |
| Tumor Histology |  |  |  | 0.24 |
| Mucinous | 10 | 1 | 9 |  |
| Signet Ring | 1 | 0 | 1 |  |
| Neither | 27 | 19 | 52 |  |
| Grade |  |  |  | 0.29 |
| Well differentiated | 0 | 0 | 1 |  |
| Moderately differentiated | 24 | 15 | 50 |  |
| Poorly differentiated | 9 | 3 | 6 |  |
| Unknown | 5 | 2 | 5 |  |
