# Supplementary material for: Src activity is modulated by oxaliplatin and correlates with outcomes after hepatectomy for metastatic colorectal cancer
Source: BMC Cancer. 2014 Sep 10;14:660. doi: 10.1186/1471-2407-14-660 (PMC4167273; doi:10.1186/1471-2407-14-660)
Supplement: Supplementary file 3 — Additional file 3: Table S3: Associations between mutation status and relative levels of activated Src and FAK. (DOCX 14 KB) [file 12885_2014_4839_MOESM3_ESM.docx]

**Additional file 3: Table S3:** Associations between mutation status and relative levels of activated Src and FAK.

|  | **Wild-Type** | **Mutated** | ***P-value*** |
| --- | --- | --- | --- |
| KRAS |  |  |  |
| pSrc/Src | 0.92 | 0.94 | 0.55 |
| pFAK/FAK | 1.00 | 0.91 | 0.22 |
| NRAS |  |  |  |
| pSrc/Src | 0.94 | 0.74 | 0.006 |
| pFAK/FAK | 1.01 | 0.55 | <0.001 |
| BRAF |  |  |  |
| pSrc/Src | 0.92 | 0.91 | 0.89 |
| pFAK/FAK | 0.96 | 1.20 | 0.17 |
| BCTTN1 |  |  |  |
| pSrc/Src | 0.94 | 0.69 | <0.001 |
| pFAK/FAK | 1.01 | 0.56 | <0.001 |
